# Supplementary figures and images for: Epidemiology of Hepatitis C virus infection among incarcerated populations in North Dakota
Source: PLoS One. 2022 Mar 29;17(3):e0266047. doi: 10.1371/journal.pone.0266047 (PMC8963564; doi:10.1371/journal.pone.0266047)

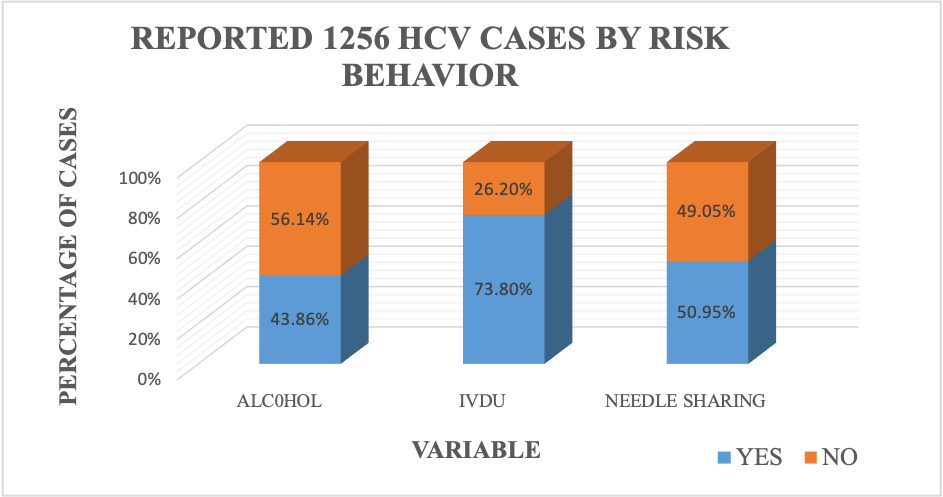

Supplement: S1 Fig — (TIFF) [file pone.0266047.s001.tiff]
