# Supplementary material for: Epidemiology of Hepatitis C virus infection among incarcerated populations in North Dakota
Source: PLoS One. 2022 Mar 29;17(3):e0266047. doi: 10.1371/journal.pone.0266047 (PMC8963564; doi:10.1371/journal.pone.0266047)
Supplement: S1 Table — (PDF) [file pone.0266047.s002.pdf]

S2 Table

	IVDU	No IVDU	
Sex			<.0001
Female	680 (26.49)	538 (11.41)	
Male	1887 (73.51)	4178 (88.59)	
Race/ethnicity			<.0001
Black	30 (1.17)	392 (8.31)	
Caucasian	1351 (52.63)	1827 (38.74)	
HIS	79 (3.08)	195 (4.13)	
NAT	533 (20.76)	551 (11.68)	
Others	574 (22.36)	1751 (37.13)	
